# An American Text-Book of Surgery, for Practitioners and Students

**Published:** 1900-01

**Authors:** 


AN AMERICAN TEXT-BOOK OF SURGERY, FOR PRACTITIONERS AND STUDENTS. By Phineas S. Connor, M.D., Frederick S. Dennis, M.D., William W. Keen, M.D., Charles B. Nancrede, M.D., Roswell Park, M.D., Lewis S. Pilcher, M.D., Nicholas Senn, M.D., Francis J. Shepard, M.D., Lewis A. Stimson, M.D., J. Collins Warren, M.D., and J. William White, M.D. Edited by William W. Keen, M.D., LL.D., and J. William White, M.D. Third edition. Thoroughly revised. Philadelphia. W. B. Saunders, 1899. Price, Cloth $7.00; Sheep or half Morocco, $8.00.

The names of the above authors should, in themselves, afford sufficient guarantee of the excellence of the work. The authors treat the subjects under "General," "Special," "Regional" and "Operative Surgery." Both principles and practice, as well as minor surgery, bacteriology, X-rays, and the latest additions to surgical knowledge of all kinds, are included. Profuse illustrations, mostly original, add much in the elucidation of the text.

